# 6-Hy­droxy-2*H*-1,3-benzodioxole-5-carbaldehyde

**DOI:** 10.1107/S1600536811037019

**Published:** 2011-09-17

**Authors:** Mehbub I. K. Momin, Neil Koorbanally, Deresh Ramjugernath, Muhammad D. Bala

**Affiliations:** a School of Chemistry, University of KwaZulu-Natal, Westville Campus, Private Bag X54001, Durban 4000, South Africa; b School of Chemical Engineering, University of KwaZulu-Natal, Private Bag X54001, Durban 4000, South Africa

## Abstract

The title compound, C_8_H_6_O_4_, crystallizes with two independent mol­ecules in the asymmetric unit. The benzodioxole ring system is almost planar in each mol­ecule, with maximum deviations of 0.008 (1) and 0.007 (1) Å. The mol­ecular structure is characterized by strong electrostatic intra­molecular O⋯O contacts [2.649 (3) Å] and intra­molecular O—H⋯O hydrogen-bonding inter­actions. Inter­molecular O⋯O inter­actions [3.001 (2) Å] are observed in the crystal structure.

## Related literature

For the preparation, see: Juhász *et al.* (2007 ▶); Akselsen *et al.* (2009 ▶). For hydrogen-bond motifs, see: Bernstein *et al.* (1995 ▶). The title compound is a starting material and an inter­mediate in the synthesis of biologically active compounds. These compounds have shown HIV-1 integrase inhibitory activity (Bailly *et al.*, 2005 ▶), dopamine D1 receptor full agonist (Cueva, *et al.* 2006 ▶) and glycogen phospho­rylase inhibitory activity (Juhász *et al.*, 2007 ▶).
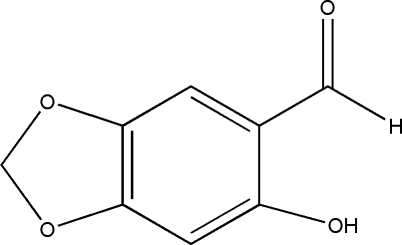


## Experimental

### 

#### Crystal data

C_8_H_6_O_4_
*M* *_r_* = 166.13
Monoclinic, 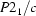

*a* = 6.4916 (3) Å
*b* = 12.8242 (7) Å
*c* = 16.7122 (8) Å
β = 96.258 (3)°
*V* = 1382.99 (12) Å^3^
*Z* = 8
Mo *K*α radiation
μ = 0.13 mm^−1^
*T* = 173 K
0.38 × 0.11 × 0.10 mm

#### Data collection

Bruker APEXII CCD diffractometer
10746 measured reflections
2708 independent reflections
1348 reflections with *I* > 2σ(*I*)
*R*_int_ = 0.081

#### Refinement

*R*[*F* ^2^ > 2σ(*F* ^2^)] = 0.045
*wR*(*F* ^2^) = 0.125
*S* = 0.94
2708 reflections
219 parameters
H-atom parameters constrained
Δρ_max_ = 0.19 e Å^−3^
Δρ_min_ = −0.31 e Å^−3^

### 

Data collection: *APEX2* (Bruker, 2009 ▶); cell refinement: *SAINT-Plus* (Bruker, 2009 ▶); data reduction: *SAINT-Plus*; program(s) used to solve structure: *SHELXS97* (Sheldrick, 2008 ▶); program(s) used to refine structure: *SHELXL97* (Sheldrick, 2008 ▶); molecular graphics: *ORTEP-3* (Farrugia, 1997 ▶); software used to prepare material for publication: *WinGX* (Farrugia, 1999 ▶).

## Supplementary Material

Crystal structure: contains datablock(s) global, I. DOI: 10.1107/S1600536811037019/qm2028sup1.cif

Structure factors: contains datablock(s) I. DOI: 10.1107/S1600536811037019/qm2028Isup2.hkl

Supplementary material file. DOI: 10.1107/S1600536811037019/qm2028Isup3.cml

Additional supplementary materials:  crystallographic information; 3D view; checkCIF report

## Figures and Tables

**Table 1:** Hydrogen-bond geometry (Å, °)

*D*—H⋯*A*	*D*—H	H⋯*A*	*D*⋯*A*	*D*—H⋯*A*
O1—H1⋯O4	0.84	1.92	2.652 (3)	146
O5—H5⋯O8	0.84	1.91	2.645 (3)	145

**Fig. 1.**
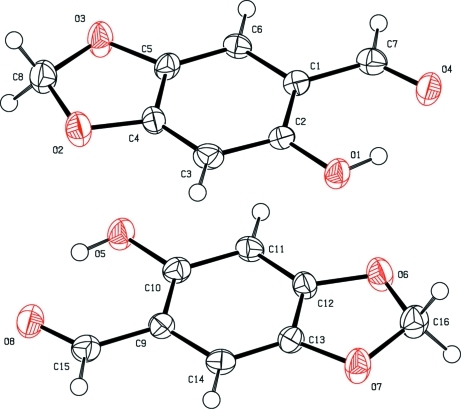
Molecular structure of the title compound with the atom numbering scheme. Displacement ellipsoids are drawn at the 50% probability level. H atoms are presented as small spheres of arbitrary radius.

## supplementary crystallographic information

### Comment

The title compound 6- hydroxybenzo[*d*][1,3]dioxole-5-carbaldehyde was obtained as an intermediate product in our research effort aimed at the total synthesis of biologically active compounds. These compounds have been used for HIV-1 integrase inhibitory activities as reported by Bailly *et al.* (2005), Dopamine D1 receptor full agonist (Cueva, *et al.* 2006) and glycogen phosphorylase inhibitory activity reported by Juhász *et al.* (2007). The compound has been previously reported by Juhász *et al.* (2007) and Akselsen *et al.* (2009) with 45% yield when it was respectively utilized as a starting material and as an intermediate in the synthesis of the biologically active compounds. However, inspite of the varied biological applications of (I) the crystal structure of the title compound has not been reported to date. The compound has two independent molecules in the asymmetric unit that are related by a crystallographic centre of inversion and a glide plane perpendicular to the (0, 1, 0) axis. The benzodioxole ring systems in the title compound are almost planar and show strong pi-pi interactions in the unit cell. The molecule is stabilized by intra-molecular hydrogen bonding contacts which are however balanced by a network of O···O electrostatic contacts that are both intra- [O1···O4 & O5···O8 = 2.649 (3) Å] and inter-molecular [O4···O8 = 3.001 (2) Å] in nature.

### Experimental

The compound 2-yydroxy-4,5-methylenedioxybenzaldehyde was synthesized by following the literature method of Akselsen *et al.* (2009). Brown crystals suitable for X-ray diffraction were grown from hexane:ethyl acetate (95:5). m.p. 125–127 °C. ^1^H NMR: δ (p.p.m.): 6.01 (2*H*,s, O–CH_2_–O); 6.46 (1*H*, s, H-5); 6.86 (1*H*, s, H-8); 9.62 (1*H*, s, CHO); 11.79 (1*H*, s, OH). ^13^C NMR: δ = 98.37, 102.15, 109.35, 113.65, 141.33, 155.17, 161.54, 193.69. HRMS m/*z* 166.0264 (calcd for C_8_H_6_O_4_: 166.0266).

### Refinement

All H-atoms were refined using a riding model, with C—H = 0.95 Å and *U*_iso_(H) = 1.2*U*_eq_(C) for aromatic, C—H = 0.99 Å and *U*_iso_(H) = 1.2*U*_eq_(C) for CH_2_.

### Crystal data

**Table:**

C_8_H_6_O_4_	*F*(000) = 688
*M**_r_* = 166.13	*D*_x_ = 1.596 Mg m^−^^3^
Monoclinic, *P*2_1_/*c*	Mo *K*α radiation, λ = 0.71073 Å
Hall symbol: -P 2ybc	Cell parameters from 1671 reflections
*a* = 6.4916 (3) Å	θ = 2.5–26.6°
*b* = 12.8242 (7) Å	µ = 0.13 mm^−^^1^
*c* = 16.7122 (8) Å	*T* = 173 K
β = 96.258 (3)°	Needle, colourless
*V* = 1382.99 (12) Å^3^	0.38 × 0.11 × 0.10 mm
*Z* = 8	

### Data collection

**Table:**

Bruker APEXII CCD diffractometer	1348 reflections with *I* > 2σ(*I*)
Radiation source: fine-focus sealed tube	*R*_int_ = 0.081
graphite	θ_max_ = 26.0°, θ_min_ = 2.0°
φ and ω scans	*h* = −7→8
10746 measured reflections	*k* = −15→15
2708 independent reflections	*l* = −18→20

### Refinement

**Table:**

Refinement on *F*^2^	Primary atom site location: structure-invariant direct methods
Least-squares matrix: full	Secondary atom site location: difference Fourier map
*R*[*F*^2^ > 2σ(*F*^2^)] = 0.045	Hydrogen site location: inferred from neighbouring sites
*wR*(*F*^2^) = 0.125	H-atom parameters constrained
*S* = 0.94	*w* = 1/[σ^2^(*F*_o_^2^) + (0.0556*P*)^2^] where *P* = (*F*_o_^2^ + 2*F*_c_^2^)/3
2708 reflections	(Δ/σ)_max_ < 0.001
219 parameters	Δρ_max_ = 0.19 e Å^−^^3^
0 restraints	Δρ_min_ = −0.31 e Å^−^^3^

### Special details

**Table:**

Geometry. All e.s.d.'s (except the e.s.d. in the dihedral angle between two l.s. planes) are estimated using the full covariance matrix. The cell e.s.d.'s are taken into account individually in the estimation of e.s.d.'s in distances, angles and torsion angles; correlations between e.s.d.'s in cell parameters are only used when they are defined by crystal symmetry. An approximate (isotropic) treatment of cell e.s.d.'s is used for estimating e.s.d.'s involving l.s. planes.
Refinement. Refinement of *F*^2^ against ALL reflections. The weighted *R*-factor *wR* and goodness of fit *S* are based on *F*^2^, conventional *R*-factors *R* are based on *F*, with *F* set to zero for negative *F*^2^. The threshold expression of *F*^2^ > σ(*F*^2^) is used only for calculating *R*-factors(gt) *etc*. and is not relevant to the choice of reflections for refinement. *R*-factors based on *F*^2^ are statistically about twice as large as those based on *F*, and *R*- factors based on ALL data will be even larger.

### Fractional atomic coordinates and isotropic or equivalent isotropic displacement parameters (Å^2^)

**Table:**

	*x*	*y*	*z*	*U*_iso_*/*U*_eq_	
C1	0.0179 (4)	0.62547 (19)	0.46651 (15)	0.0211 (6)	
C2	0.2306 (4)	0.6144 (2)	0.49173 (15)	0.0221 (6)	
C3	0.3046 (4)	0.6148 (2)	0.57346 (15)	0.0272 (7)	
H3	0.4474	0.6059	0.5915	0.033*	
C4	0.1604 (4)	0.6288 (2)	0.62576 (14)	0.0233 (6)	
C5	−0.0500 (4)	0.6406 (2)	0.60194 (15)	0.0230 (6)	
C6	−0.1269 (4)	0.63872 (19)	0.52350 (15)	0.0225 (6)	
H6	−0.2710	0.6459	0.5073	0.027*	
C7	−0.0596 (4)	0.6209 (2)	0.38261 (16)	0.0265 (7)	
H7	−0.2052	0.6268	0.3691	0.032*	
C8	−0.0031 (5)	0.6428 (2)	0.73701 (16)	0.0328 (7)	
H8A	−0.0324	0.5811	0.7695	0.039*	
H8B	−0.0048	0.7056	0.7714	0.039*	
C9	0.4907 (4)	0.6181 (2)	0.12152 (15)	0.0240 (6)	
C10	0.2786 (4)	0.6096 (2)	0.09607 (15)	0.0247 (6)	
C11	0.2024 (4)	0.6141 (2)	0.01456 (14)	0.0248 (7)	
H11	0.0591	0.6072	−0.0034	0.030*	
C12	0.3487 (4)	0.6294 (2)	−0.03776 (15)	0.0238 (6)	
C13	0.5577 (4)	0.6389 (2)	−0.01390 (15)	0.0236 (6)	
C14	0.6353 (4)	0.6332 (2)	0.06454 (15)	0.0244 (6)	
H14	0.7797	0.6389	0.0807	0.029*	
C15	0.5696 (4)	0.6118 (2)	0.20578 (16)	0.0283 (7)	
H15	0.7151	0.6174	0.2196	0.034*	
C16	0.5093 (4)	0.6575 (2)	−0.14766 (16)	0.0316 (7)	
H16A	0.5389	0.6056	−0.1887	0.038*	
H16B	0.5077	0.7277	−0.1724	0.038*	
O1	0.3710 (3)	0.60201 (16)	0.43841 (10)	0.0301 (5)	
H1	0.3094	0.5985	0.3916	0.045*	
O2	0.1955 (3)	0.63205 (16)	0.70789 (10)	0.0353 (5)	
O3	−0.1564 (3)	0.65199 (16)	0.66900 (10)	0.0343 (5)	
O4	0.0485 (3)	0.60984 (15)	0.32659 (10)	0.0314 (5)	
O5	0.1369 (3)	0.59693 (17)	0.14948 (11)	0.0336 (5)	
H5	0.1983	0.5920	0.1962	0.050*	
O6	0.3117 (3)	0.63578 (15)	−0.11966 (11)	0.0331 (5)	
O7	0.6644 (3)	0.65268 (16)	−0.08064 (10)	0.0330 (5)	
O8	0.4600 (3)	0.59961 (16)	0.26055 (11)	0.0357 (5)	

### Atomic displacement parameters (Å^2^)

**Table:**

	*U*^11^	*U*^22^	*U*^33^	*U*^12^	*U*^13^	*U*^23^
C1	0.0199 (15)	0.0198 (14)	0.0233 (15)	0.0015 (12)	0.0015 (12)	0.0026 (11)
C2	0.0210 (15)	0.0221 (15)	0.0235 (15)	−0.0035 (12)	0.0041 (12)	0.0004 (11)
C3	0.0176 (15)	0.0323 (17)	0.0310 (17)	−0.0011 (13)	0.0000 (13)	0.0009 (12)
C4	0.0250 (16)	0.0266 (15)	0.0182 (15)	−0.0003 (13)	0.0010 (12)	−0.0011 (11)
C5	0.0215 (15)	0.0252 (15)	0.0238 (16)	0.0018 (13)	0.0088 (12)	−0.0026 (12)
C6	0.0158 (14)	0.0247 (15)	0.0262 (15)	0.0011 (13)	−0.0016 (12)	0.0006 (12)
C7	0.0236 (16)	0.0246 (16)	0.0302 (17)	0.0002 (13)	−0.0020 (13)	0.0032 (12)
C8	0.0281 (17)	0.0458 (19)	0.0248 (16)	0.0026 (15)	0.0042 (13)	−0.0019 (14)
C9	0.0256 (15)	0.0238 (15)	0.0225 (15)	0.0029 (14)	0.0014 (12)	−0.0019 (12)
C10	0.0223 (16)	0.0248 (15)	0.0276 (16)	0.0002 (13)	0.0055 (12)	0.0007 (12)
C11	0.0154 (14)	0.0319 (17)	0.0263 (16)	0.0005 (13)	−0.0009 (12)	0.0011 (12)
C12	0.0261 (16)	0.0254 (15)	0.0191 (14)	0.0010 (13)	−0.0006 (12)	0.0000 (12)
C13	0.0223 (15)	0.0252 (15)	0.0238 (16)	−0.0007 (13)	0.0049 (12)	0.0015 (12)
C14	0.0156 (14)	0.0278 (16)	0.0297 (16)	−0.0017 (13)	0.0020 (12)	−0.0001 (12)
C15	0.0228 (15)	0.0339 (17)	0.0282 (16)	0.0023 (14)	0.0024 (13)	−0.0019 (13)
C16	0.0290 (17)	0.0385 (17)	0.0281 (17)	−0.0045 (15)	0.0073 (14)	0.0025 (13)
O1	0.0191 (11)	0.0496 (13)	0.0217 (11)	0.0002 (10)	0.0035 (8)	0.0014 (10)
O2	0.0234 (12)	0.0598 (14)	0.0224 (11)	0.0034 (10)	0.0015 (9)	−0.0048 (10)
O3	0.0238 (11)	0.0573 (15)	0.0225 (11)	0.0067 (10)	0.0053 (9)	−0.0045 (10)
O4	0.0321 (12)	0.0414 (12)	0.0217 (11)	0.0012 (10)	0.0068 (9)	0.0020 (9)
O5	0.0208 (11)	0.0557 (14)	0.0252 (11)	−0.0006 (10)	0.0061 (9)	0.0015 (10)
O6	0.0271 (12)	0.0484 (13)	0.0232 (11)	−0.0025 (10)	−0.0003 (9)	0.0039 (9)
O7	0.0257 (12)	0.0508 (14)	0.0231 (11)	−0.0075 (10)	0.0051 (9)	0.0011 (9)
O8	0.0335 (13)	0.0493 (14)	0.0250 (11)	0.0020 (11)	0.0062 (9)	−0.0010 (10)

### Geometric parameters (Å, °)

**Table:**

C1—C2	1.407 (4)	C9—C14	1.422 (3)
C1—C6	1.419 (3)	C9—C15	1.447 (4)
C1—C7	1.438 (4)	C10—O5	1.360 (3)
C2—O1	1.351 (3)	C10—C11	1.399 (3)
C2—C3	1.398 (3)	C11—C12	1.373 (4)
C3—C4	1.360 (3)	C11—H11	0.9500
C3—H3	0.9500	C12—O6	1.366 (3)
C4—O2	1.367 (3)	C12—C13	1.378 (4)
C4—C5	1.389 (4)	C13—C14	1.354 (4)
C5—C6	1.351 (3)	C13—O7	1.387 (3)
C5—O3	1.387 (3)	C14—H14	0.9500
C6—H6	0.9500	C15—O8	1.229 (3)
C7—O4	1.238 (3)	C15—H15	0.9500
C7—H7	0.9500	C16—O7	1.423 (3)
C8—O3	1.432 (3)	C16—O6	1.440 (3)
C8—O2	1.433 (3)	C16—H16A	0.9900
C8—H8A	0.9900	C16—H16B	0.9900
C8—H8B	0.9900	O1—H1	0.8400
C9—C10	1.400 (4)	O5—H5	0.8400
			
C2—C1—C6	120.8 (2)	O5—C10—C11	116.8 (2)
C2—C1—C7	121.0 (2)	O5—C10—C9	121.5 (2)
C6—C1—C7	118.2 (3)	C11—C10—C9	121.6 (2)
O1—C2—C3	117.4 (2)	C12—C11—C10	115.5 (3)
O1—C2—C1	121.7 (2)	C12—C11—H11	122.3
C3—C2—C1	120.9 (2)	C10—C11—H11	122.3
C4—C3—C2	116.2 (3)	O6—C12—C11	126.0 (3)
C4—C3—H3	121.9	O6—C12—C13	110.2 (2)
C2—C3—H3	121.9	C11—C12—C13	123.8 (2)
C3—C4—O2	126.7 (3)	C14—C13—C12	121.7 (2)
C3—C4—C5	123.7 (2)	C14—C13—O7	128.3 (3)
O2—C4—C5	109.6 (2)	C12—C13—O7	109.9 (2)
C6—C5—O3	128.5 (3)	C13—C14—C9	116.9 (3)
C6—C5—C4	121.6 (2)	C13—C14—H14	121.5
O3—C5—C4	109.9 (2)	C9—C14—H14	121.5
C5—C6—C1	116.8 (3)	O8—C15—C9	124.0 (3)
C5—C6—H6	121.6	O8—C15—H15	118.0
C1—C6—H6	121.6	C9—C15—H15	118.0
O4—C7—C1	125.1 (3)	O7—C16—O6	108.3 (2)
O4—C7—H7	117.5	O7—C16—H16A	110.0
C1—C7—H7	117.5	O6—C16—H16A	110.0
O3—C8—O2	108.1 (2)	O7—C16—H16B	110.0
O3—C8—H8A	110.1	O6—C16—H16B	110.0
O2—C8—H8A	110.1	H16A—C16—H16B	108.4
O3—C8—H8B	110.1	C2—O1—H1	109.5
O2—C8—H8B	110.1	C4—O2—C8	106.6 (2)
H8A—C8—H8B	108.4	C5—O3—C8	105.6 (2)
C10—C9—C14	120.4 (2)	C10—O5—H5	109.5
C10—C9—C15	121.6 (2)	C12—O6—C16	105.8 (2)
C14—C9—C15	118.0 (3)	C13—O7—C16	105.4 (2)
			
C6—C1—C2—O1	−179.8 (2)	C10—C11—C12—O6	179.6 (2)
C7—C1—C2—O1	1.9 (4)	C10—C11—C12—C13	0.6 (4)
C6—C1—C2—C3	0.8 (4)	O6—C12—C13—C14	−178.7 (2)
C7—C1—C2—C3	−177.5 (2)	C11—C12—C13—C14	0.4 (4)
O1—C2—C3—C4	179.1 (2)	O6—C12—C13—O7	0.4 (3)
C1—C2—C3—C4	−1.4 (4)	C11—C12—C13—O7	179.5 (2)
C2—C3—C4—O2	180.0 (2)	C12—C13—C14—C9	−0.8 (4)
C2—C3—C4—C5	1.1 (4)	O7—C13—C14—C9	−179.6 (2)
C3—C4—C5—C6	0.0 (4)	C10—C9—C14—C13	0.1 (4)
O2—C4—C5—C6	−179.0 (2)	C15—C9—C14—C13	−179.9 (2)
C3—C4—C5—O3	179.2 (3)	C10—C9—C15—O8	−0.5 (4)
O2—C4—C5—O3	0.2 (3)	C14—C9—C15—O8	179.5 (3)
O3—C5—C6—C1	−179.8 (2)	C3—C4—O2—C8	−177.1 (3)
C4—C5—C6—C1	−0.7 (4)	C5—C4—O2—C8	2.0 (3)
C2—C1—C6—C5	0.3 (4)	O3—C8—O2—C4	−3.3 (3)
C7—C1—C6—C5	178.7 (2)	C6—C5—O3—C8	176.9 (3)
C2—C1—C7—O4	−1.8 (4)	C4—C5—O3—C8	−2.2 (3)
C6—C1—C7—O4	179.8 (3)	O2—C8—O3—C5	3.4 (3)
C14—C9—C10—O5	−178.8 (3)	C11—C12—O6—C16	177.0 (3)
C15—C9—C10—O5	1.2 (4)	C13—C12—O6—C16	−3.9 (3)
C14—C9—C10—C11	0.9 (4)	O7—C16—O6—C12	6.0 (3)
C15—C9—C10—C11	−179.1 (3)	C14—C13—O7—C16	−177.7 (3)
O5—C10—C11—C12	178.5 (2)	C12—C13—O7—C16	3.4 (3)
C9—C10—C11—C12	−1.2 (4)	O6—C16—O7—C13	−5.7 (3)

### Hydrogen-bond geometry (Å, °)

**Table:**

*D*—H···*A*	*D*—H	H···*A*	*D*···*A*	*D*—H···*A*
O1—H1···O4	0.84	1.92	2.652 (3)	146.
O5—H5···O8	0.84	1.91	2.645 (3)	145.
